# Supplementary material for: Improving the Power of Structural Variation Detection by Augmenting the Reference
Source: PLoS One. 2015 Aug 31;10(8):e0136771. doi: 10.1371/journal.pone.0136771 (PMC4556445; doi:10.1371/journal.pone.0136771)
Supplement: S1 File — Fig A shows Venter Novel Alleles locations. Fig B shows the proportion of validated VNA sites that have a VNA allele, per individual, segregated by population (as judged by the validation classifier). Fig C shows the relationship of accuracy to VNA size. Table A shows a description of dataset. Table B shows the pipeline accuracies. Table C shows the validation dataset. (DOCX) [file pone.0136771.s001.docx]

IMPROVING POWER OF STRUCTURAL VARIATION DETECTION BY AUGMENTING THE REFERENCE

Jan Schroeder, Santhosh Girirajan, Anthony T. Papenfuss, Paul Medvedev

S1 File: Supplementary Tables and Figures

Contents:

| Supplementary Figure/Table | Title |
| --- | --- |
| Supplementary Figure A | Venter Novel Allele locations |
| Supplementary Figure B | Number of VNAs per individual, segregated by population |
| Supplementary Figure C | Relationship of accuracy to VNA size. |
| Supplementary Table A | Description of dataset |
| Supplementary Table B | Pipeline Accuracies |
| Supplementary Table C | Validation dataset |

**Supplementary Figure A:** Venter Novel Alleles locations. We show the location of the VNAs and the genes they overlap. The figure is generating using the PhenoGram software [15].

**
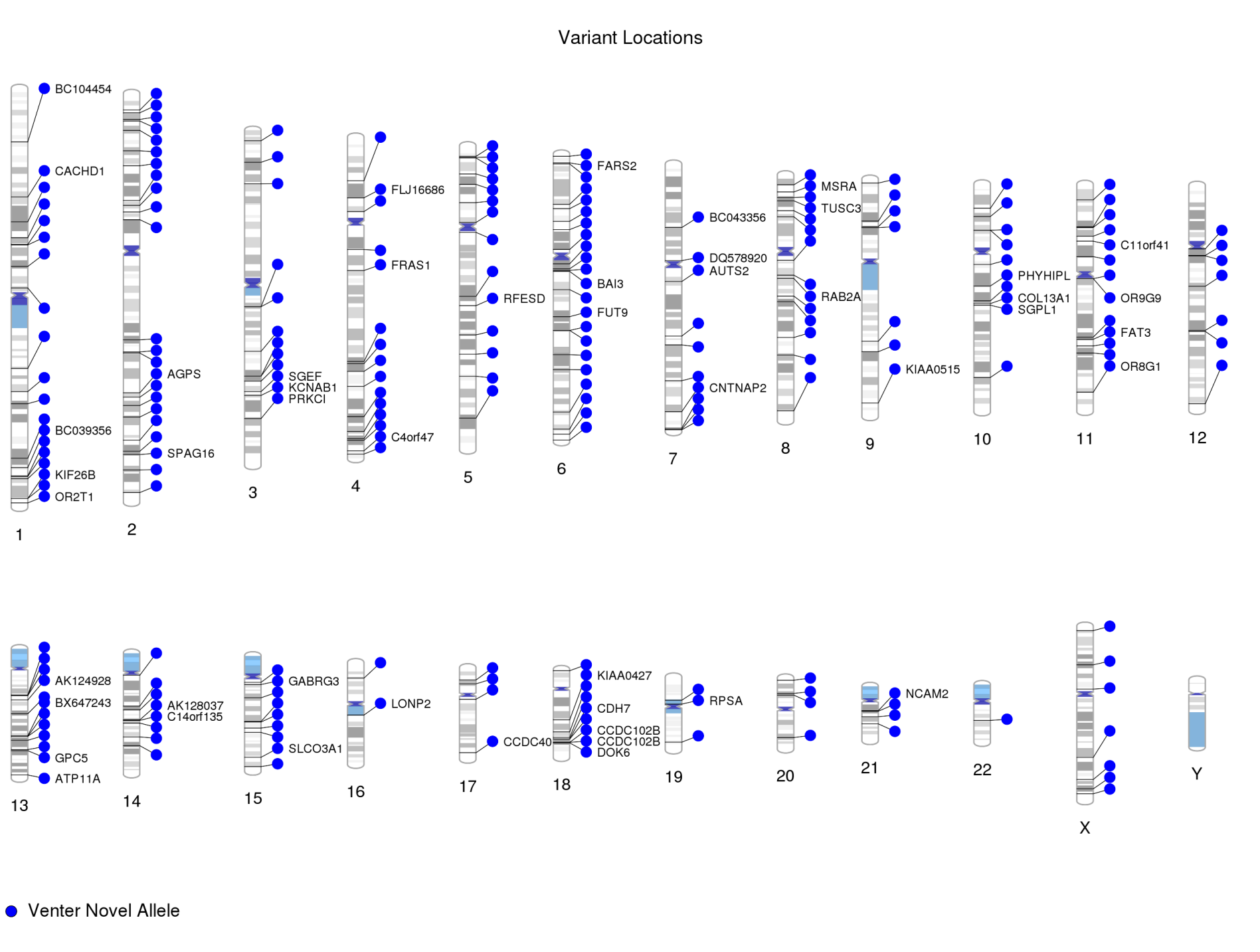
**

**Supplementary Figure B:** Proportion of validated VNA sites that have a VNA allele, per individual, segregated by population (as judged by the validation classifier).


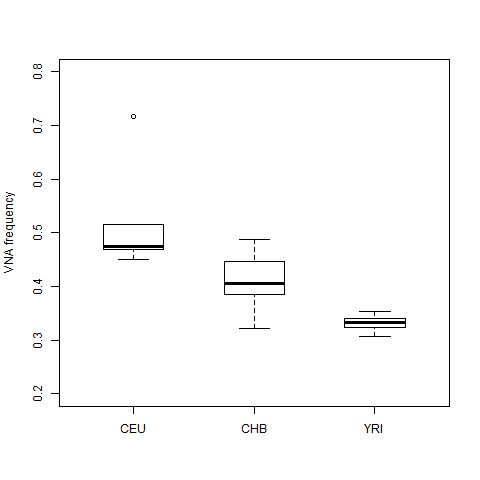


**Supplementary Figure C:** Relationship of accuracy to VNA size. The plot demonstrates the accuracy (y-axis), over all 16 samples, for VNAs within a given size range (x-axis). The figure also shows the number of VNAs that fall into a given size range.


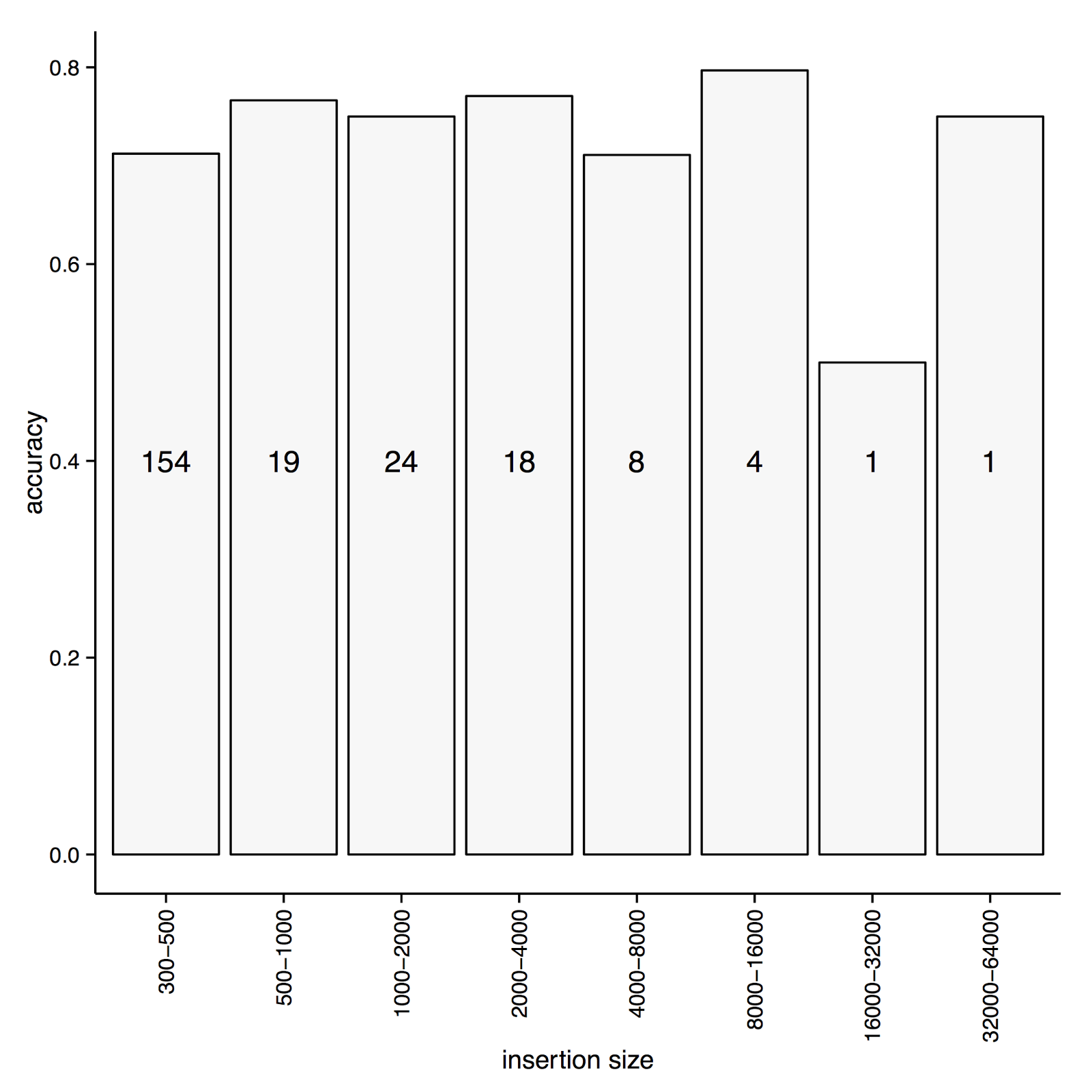


**Supplementary Table A:** Description of dataset.

| Pop | Sample | Accession Numbers | Read Coverage |
| --- | --- | --- | --- |
| CEU | NA07037 | ERR257983, ERR260404 | 16.3 |
|  | NA10847 | ERR257984, ERR260405 | 8.8 |
|  | NA12716 | ERR257986 | 5.8 |
|  | NA12717 | ERR257987 | 6.3 |
|  | NA12750 | ERR257985 | 7.1 |
|  | NA12882 | ERR091575 | 15.0 |
| YRI | NA18489 | SRR788627, SRR788642, SRR788646, SRR788649, SRR788657 | 3.1 |
|  | NA18507 | SRR034968, SRR034969, SRR034970, SRR034971, SRR034972, SRR034973, SRR034974, SRR034975 | 11.0 |
|  | NA18519 | SRR788625, SRR788626, SRR788629, SRR788637, SRR788639, SRR788641 | 3.8 |
|  | NA18881 | ERR257965 | 6.7 |
|  | NA18915 | ERR257966, ERR260397 | 7.7 |
| CHB | NA18615 | ERR009359, ERR009360, ERR009364, ERR009366, ERR009382, ERR009395, ERR009398 | 6.6 |
|  | NA18616 | ERR009361, ERR009370, ERR009387, ERR009389, ERR009396, ERR009399 | 7.3 |
|  | NA18617 | ERR009367, ERR009379, ERR009381, ERR009388, ERR009390, ERR009393, ERR009394 | 7.7 |
|  | NA18628 | ERR009371, ERR009372, ERR009373, ERR009383, ERR009385, ERR009386, ERR009392 | 7.7 |
|  | NA18631 | ERR009363, ERR009365, ERR009376, ERR009377, ERR009384 | 5.7 |

**Supplementary Table B:** Pipeline accuracies. This table shows the accuracy of our ref+ and GRC pipelines for each of the individuals. For each sample, the validated sites are those for which our validation classifier finds evidence for at least one of the alleles (GRC or VNA).

| Pop | Sample | VNA  frequency | Num  Validated  Sites | GRC pipeline | | | | | | | ref+ pipeline | | | | | | |
| --- | --- | --- | --- | --- | --- | --- | --- | --- | --- | --- | --- | --- | --- | --- | --- | --- | --- |
|  |  |  |  | TP | FP | FN | TN | FDR | Sensitivity | Accuracy | TP | FP | FN | TN | FDR | Sensitivity | Accuracy |
| CEU | NA07037 | 0.469 | 228 | 5 | 3 | 145 | 75 | 0.38 | 0.03 | 0.35 | 107 | 4 | 43 | 74 | 0.04 | 0.71 | 0.79 |
|  | NA10847 | 0.516 | 221 | 8 | 0 | 144 | 69 | 0.00 | 0.05 | 0.35 | 96 | 6 | 56 | 63 | 0.06 | 0.63 | 0.72 |
|  | NA12716 | 0.468 | 205 | 4 | 2 | 117 | 82 | 0.33 | 0.03 | 0.42 | 94 | 12 | 27 | 72 | 0.11 | 0.78 | 0.81 |
|  | NA12717 | 0.717 | 161 | 1 | 2 | 121 | 37 | 0.67 | 0.01 | 0.24 | 99 | 5 | 23 | 34 | 0.05 | 0.81 | 0.83 |
|  | NA12750 | 0.450 | 221 | 5 | 1 | 119 | 96 | 0.17 | 0.04 | 0.46 | 94 | 9 | 30 | 88 | 0.09 | 0.76 | 0.82 |
|  | NA12882 | 0.480 | 229 | 13 | 1 | 159 | 56 | 0.07 | 0.08 | 0.30 | 121 | 5 | 51 | 52 | 0.04 | 0.70 | 0.76 |
| YRI | NA18489 | 0.306 | 198 | 11 | 6 | 63 | 118 | 0.35 | 0.15 | 0.65 | 67 | 59 | 7 | 65 | 0.47 | 0.91 | 0.67 |
|  | NA18507 | 0.353 | 214 | 3 | 1 | 120 | 90 | 0.25 | 0.02 | 0.43 | 77 | 6 | 46 | 85 | 0.07 | 0.63 | 0.76 |
|  | NA18519 | 0.333 | 203 | 8 | 15 | 83 | 97 | 0.65 | 0.09 | 0.52 | 72 | 32 | 19 | 80 | 0.31 | 0.79 | 0.75 |
|  | NA18881 | 0.341 | 208 | 8 | 3 | 77 | 120 | 0.27 | 0.09 | 0.62 | 65 | 16 | 20 | 107 | 0.20 | 0.76 | 0.83 |
|  | NA18915 | 0.323 | 195 | 6 | 4 | 70 | 115 | 0.40 | 0.08 | 0.62 | 55 | 12 | 21 | 107 | 0.18 | 0.72 | 0.83 |
| CHB | NA18615 | 0.405 | 152 | 4 | 1 | 65 | 82 | 0.20 | 0.06 | 0.57 | 63 | 20 | 6 | 63 | 0.24 | 0.91 | 0.83 |
|  | NA18616 | 0.487 | 196 | 16 | 1 | 97 | 82 | 0.06 | 0.14 | 0.50 | 97 | 19 | 16 | 64 | 0.16 | 0.86 | 0.82 |
|  | NA18617 | 0.322 | 169 | 11 | 7 | 54 | 97 | 0.39 | 0.17 | 0.64 | 55 | 15 | 10 | 89 | 0.21 | 0.85 | 0.85 |
|  | NA18628 | 0.447 | 178 | 12 | 3 | 80 | 83 | 0.20 | 0.13 | 0.53 | 74 | 6 | 18 | 80 | 0.08 | 0.80 | 0.87 |
|  | NA18631 | 0.385 | 157 | 11 | 11 | 59 | 76 | 0.50 | 0.16 | 0.55 | 57 | 19 | 13 | 68 | 0.25 | 0.81 | 0.80 |

**Supplementary Table C:** Validation dataset

| Pop | Sample | Read Coverage | Accession Numbers |
| --- | --- | --- | --- |
| CEU | NA07037 | 26.4 | ERR0010[69-76], ERR00142[3-7], ERR00156[3-7], ERR00213[1-7],  ERR00[2394-2400], ERR00245[3-4], ERR002988, ERR034542, ERR257983, ERR260404 |
|  | NA10847 | 15.4 | ERR0005[53-60], ERR257984, ERR260405 |
|  | NA12716 | 12.8 | ERR0005[69-76], ERR257986 |
|  | NA12717 | 12.6 | ERR0005[77-84], ERR257987 |
|  | NA12750 | 14.7 | ERR0005[85-92], ERR257985, SRR077449, SRR081238 |
|  | NA12882 | 15.0 | ERR091575 |
| YRI | NA18489 | 15.9 | SRR0032[58-67], SRR018110, SRR02046[6-7], SRR027536, SRR100025, SRR7886[27,42,46,49,57] |
|  | NA18507 | 12.0 | SRR0349[68-75] |
|  | NA18519 | 17.7 | SRR0033[47-53], SRR0063[57-62], SRR014015, SRR01811[2-4], SRR020484, SRR027539, SRR0848[74-75,77-78,84,92-93,95-96],  SRR0849[00,02,05,07-09,11,14,18-19,25,29-31,35,40,49,52,60,69,72,75-76,78-79,84,86,88,90,92,94-95,97],  SRR0850[03,07-08,14,18,19,22-23,25,31,33,38-40,42,44,48,50-51,55,60-61],  SRR0985[11,13,17,19,21-22,29,32], SRR7886[25-26,29,37,39,41] |
|  | NA18881 | 12.8 | ERR25094[8-9], ERR257965 |
|  | NA18915 | 11.4 | ERR25095[0-1], ERR257966, ERR260397 |
| CHB | NA18615 | 6.6 | ERR0093[59-60,64,66,82,95,98], SRR09664[5-6] |
|  | NA18616 | 14.6 | ERR0093[61,70,78,87,89,96,99], SRR098336 |
|  | NA18617 | 7.7 | ERR0093[67,79,81,88,90,93-94] |
|  | NA18628 | 7.7 | ERR0093[71-73,83,85-86,92] |
|  | NA18631 | 12.8 | ERR0093[63,65,76-77,84], SRR098335 |
